# Oocyte-specific deletion of *furin* leads to female infertility by causing early secondary follicle arrest in mice

**DOI:** 10.1038/cddis.2017.231

**Published:** 2017-06-01

**Authors:** Tie-Gang Meng, Meng-Wen Hu, Xue-Shan Ma, Lin Huang, Qiu-Xia Liang, Yue Yuan, Yi Hou, Hongmei Wang, Heide Schatten, Zhen-Bo Wang, Qing-Yuan Sun

**Affiliations:** 1 State Key Laboratory of Stem Cell and Reproductive Biology, Institute of Zoology, Chinese Academy of Sciences, Beijing 100101, China; 2 University of Chinese Academy of Sciences, Beijing 100101, China; 3 Department of Veterinary Pathobiology, University of Missouri, Columbia, MO 65211, USA

## Abstract

The process of follicular development involves communications between oocyte and surrounding granulosa cells. FURIN is a member of the family of proprotein convertases that is involved in the activation of a large number of zymogens and proproteins by cleavage at its recognition motif. To investigate the functions of FURIN in female fertility, *furin*^*flox/flox*^ (*fur*^*fl/fl*^) mice were crossed with *Zp3-Cre* mice and *Gdf9-Cre*, respectively, to achieve oocyte-specific disruption of FURIN. Here we report for the first time that FURIN is dispensable for primordial follicle maintenance and activation but important for early secondary follicular development, as ablation of FURIN in oocytes caused failure of follicle development beyond the type 4 and/or 5a follicles in mutant mice, resulting in increased number of early secondary follicles and the severely decreased number of mature follicles, thus anovulation and infertility. We also found that the developmental arrest of early secondary follicles might be rooted in the loss of the mature form of ADAMTS1 (85-kDa prodomain truncated) and compromised proliferation of granulosa cells in mutant mice. Taken together, our data highlight the importance of FURIN in follicle development beyond the early secondary follicle stage and indicate that compromised FURIN function leads to follicular dysplasia and female infertility in mice.

In most cases, female infertility can be caused in many processes, including folliculogenesis, ovulation, early embryogenesis, implantation, and so on. However, premature ovarian insufficiency or premature ovarian failure with defects in follicular development in the ovary has become the main cause of female fertility, which troubles nearly 1% women under 40 years old.^1, 2, 3, 4^ The follicle, which consists of an oocyte surrounded by one or more layers of granulosa cells, is the functional content of the ovary. Follicles are distinguished as primordial follicles, primary follicles, secondary follicles and antral follicles based on the developmental stage. According to the well-accepted standards established by Pederson and Peters,^5^ the secondary follicles can be divided into early secondary and late secondary follicles according to the number of granulosa layers.^6^

Many proteins are synthesized as proproteins; FURIN has a variety of proproteins as its substrates.^7, 8, 9, 10, 11^ FURIN has been shown to have multiple functions on the proteolytic cleavage of HIV envelope polyprotein precursor gp160 to gp120 and gp41 before viral assembly, and it participates in cleaving other proteins into their mature/active forms.^12, 13, 14, 15, 16^ FURIN is ubiquitously expressed and its null mutant mice die by embryonic day 11.5.^17^ Conditional knockout model shows that FURIN has limited redundancy in mouse liver^18^ and FURIN is involved in granular acidification in endocrine pancreas by processing pro-AC45.^19^ Further, a previous report shows that in T cells without FURIN, ADAMTS6 is downregulated, which is in keeping with the theory that the PCSK-targeted molecules are coordinately regulated by their converting enzyme.^20^ In spite of the increasing number of reports about FURIN, the function of *furin* in folliculogenesis and female fertility is unknown.

*ADAMTS-1* (A disintegrin-like and metalloproteinase with thrombospondin type I motifs-1) is a metalloprotease of the family of genes that contain metalloprotease, disintegrin and thrombospondin-like motif.^21, 22^ The human ortholog of *ADAMTS-1* is named *METH-1*. The ADAMTS1 is synthesized as zymogen that is 110 kDa.^23^ The mouse *ADAMTS-1* gene was first described as a transcript, which is highly expressed in a cachexigenic colon tumor cell line.^24^ ADAMTS1 is implicated in many physiological processes. For example, ADAMTS1 has a negative role in angiogenesis, as proved by a previous study.^22, 25^ Furthermore, Shindo *et al.*^26^ reported that *ADAMTS-1*-null mice showed renal anomalies and female infertility. Subsequently, it was shown that ADAMTS1 participated in normal follicular development, in the ovulatory process, and in the organization of the medullary vascular network in the ovary.^27^ The number of follicles at type 5b (late preantral) and later stages was markedly reduced in *ADAMTS-1*-null mice. On the other hand, a previous study demonstrated that the cleavage of the prodomain from the ADAMTS1 precursor was impaired in the FURIN-deficient cell line.^28^ Histological examination shows that ADAMTS1 is necessary for maintaining normal granulosa cell layers in follicles.^27^

The aim of our present study was to determine the roles of FURIN in oogenesis and embryo development, and we adopted the *Gdf9-Cre/loxP* system and *Zp3-Cre/loxP* system, in which *furin* was selectively deleted in mouse oocytes of primordial follicles and primary follicles. We found that *furin* deletion caused female infertility and follicle arrest before the type 4 and/ 5a stage by causing failure of pro-ADAMTS1 proteolytic processing to ADAMTS1 by FURIN.

## Results

### Localization and oocyte-specific deletion of *furin*

**Figure 1:**
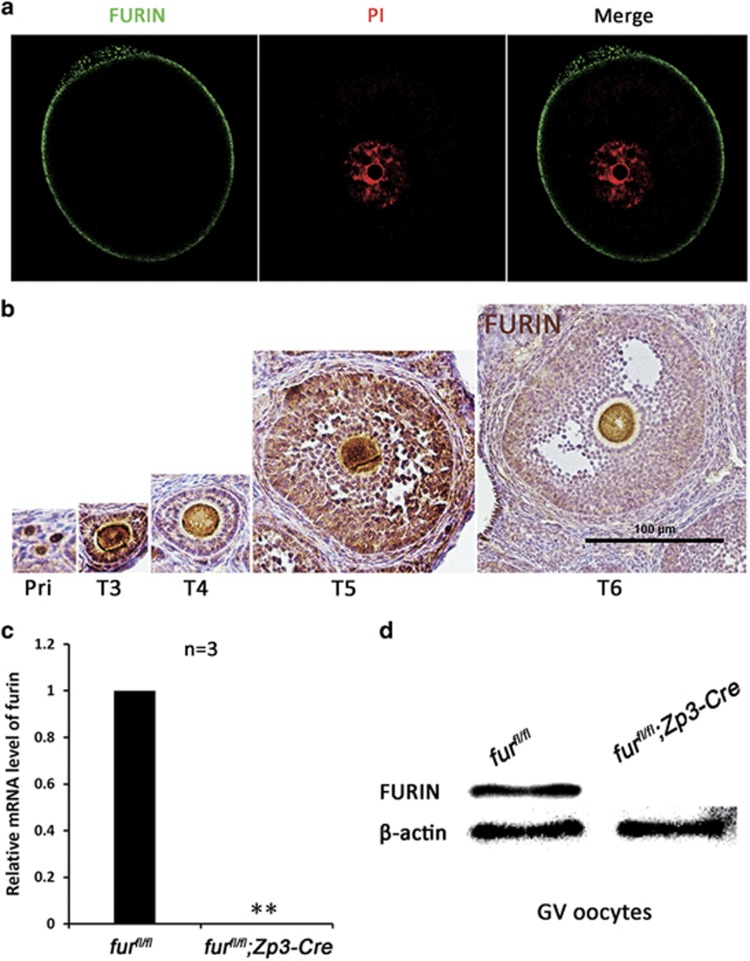
Localization and oocyte-specific deletion of *furin*. (**a**) Representative images of subcellular localization of FURIN in GV oocytes. Oocytes were immunolabeled with FURIN antibody (green) and counterstained with propidium iodide (PI) (red) for DNA. GV, germinal vesicle. Scale bar=20 *μ*m. (**b**) FURIN IHC staining showing the localization during follicular development in the mouse ovary. Scale bar=100 *μ*m. (**c**) qRT-PCR showing *furin* mRNA level in *fur*^*fl/fl*^ and *fur*^*fl/fl*^*; Zp3-Cre* oocytes, respectively (*n*=3 for each genotype). **P< 0.01. (**d**) Western blot analysis of protein level in *fur*^*fl/fl*^ and *fur*^*fl/fl*^*; Zp3-Cre* oocytes. Level of *β*-actin was used as an internal control. A total of 250 oocytes were used for each lane

To study the physiological roles of FURIN during oogenesis and embryogenesis, we first carried out immunofluorescent staining experiments by using an antibody against FURIN to show the subcellular localization of FURIN. As shown in Figure 1a, FURIN protein was localized in the cell membrane in GV oocyte. Meanwhile, immunohistochemical (IHC) analysis showed that FURIN was expressed in oocytes from the primordial follicles to mature follicles (Figure 1b).

FURIN is ubiquitously expressed in mice, and its null mutant mice die by embryonic day 11.5. To investigate the *in vivo* function of FURIN, we adopted the Cre-loxP system to achieve oocyte-specific disruption of FURIN from primary follicle stage. The mutant mice, in which exon 2 of the *furin* gene is targeted,^18^ was generated by crossing FURIN^*flox/flox*^ (*fur*^*fl/fl*^) mice with Zp3-Cre transgenic mice, which expressed Zp3 promoter-meditated Cre recombinase in oocytes of primary follicles after postnatal day 5 and in later developmental stages.^29^ Quantitative real time-PCR (qRT-PCR) test showed that the *furin* mRNA was successfully disrupted in *fur*^*fl/fl*^*;Zp3-Cre* oocytes (Figure 1c), and FURIN protein was absent in *fur*^*fl/fl*^*;Zp3-Cre* oocytes as shown by western blot analysis (Figure 1d).

### Deletion of *furin* in oocytes causes anovulation and infertility

**Figure 2:**
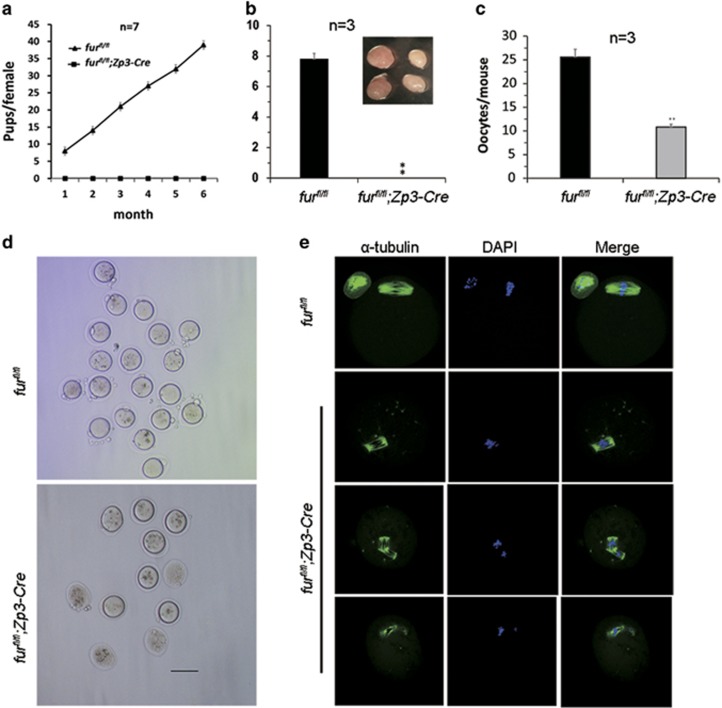
Deletion of *furin* leads to anovulation in *fur*^*fl/fl*^*; Zp3-Cre* female mice. (**a**) Breeding assays showed complete infertility of the female *fur*^*fl/fl*^*; Zp3-Cre* mice. Continuous breeding assessment showed the cumulative number of progeny per female *fur*^*fl/fl*^ and *fur*^*fl/fl*^*; Zp3-Cre* mouse for 6 months. At least seven mice of each genotype were used. (**b**) Natural ovulation assay showed decreased number of oocytes in *fur*^*fl/fl*^*; Zp3-Cre* female mice. Image in panel a shows the size of the ovary. (**c**) Superovulation assay of the number of ovulated oocytes from 6-week-old mice. Data are shown as mean±SEM. **P<0.01. (**d**) MII oocytes superovulated from *fur*^*fl/fl*^ and *fur*^*fl/fl*^*; Zp3-Cre* mouse ovaries. Scale bar=100 *μ*m. (**e**) Superovulated MII oocytes from *fur*^*fl/fl*^ and *fur*^*fl/fl*^*;Zp3-Cre* mice were fixed and double-stained for *α*-tubulin (green) and 4',6-diamidino-2-phenylindole (DAPI) (blue). MII oocytes from *fur*^*fl/fl*^*;Zp3-Cre* mice showed degenerated first polar body and abnormal spindle. All of the experiments were repeated at least three times, and representative results are shown

To identify the effect of oocyte-specific ablation of FURIN in female fertility, we carried out a breeding assay by crossing *fur*^*fl/fl*^ or *fur*^*fl/fl*^*;Zp3-Cre* female mice with fertile males for 6 months. As shown in Figure 2a, female *fur*^*fl/fl*^*;Zp3-Cre* mice were completely infertile, while the *fur*^*fl/fl*^ mice possessed normal fertility. The mutant mice (postnatal day 42) had smaller ovaries and failed to ovulate during a natural ovulation assay (Figure 2b). We speculated that the follicular development was abnormal in *fur*^*fl/fl*^*;Zp3-Cre* mice. When we carried out superovulation to induce ovulation with exogenous PMSG and hCG, the mutant mice at the age of 6 weeks ovulated fewer oocytes than the control (Figure 2c). The MII oocytes collected from mutant mice after superovulation displayed poor quality, showing degenerated polar bodies and severely abnormal spindle (Figure 2d). Further immunofluorescent staining showed abnormal spindles in *fur*^*fl/fl*^*;Zp3-Cre* oocytes (Figure 2e). Importantly, the mutant mice at the age of 8 weeks and older could not be induced to ovulate anymore (data not shown).

### Oocyte-specific deletion of *furin* causes significant decrease of late growing follicles but increase of early secondary follicles

**Figure 3:**
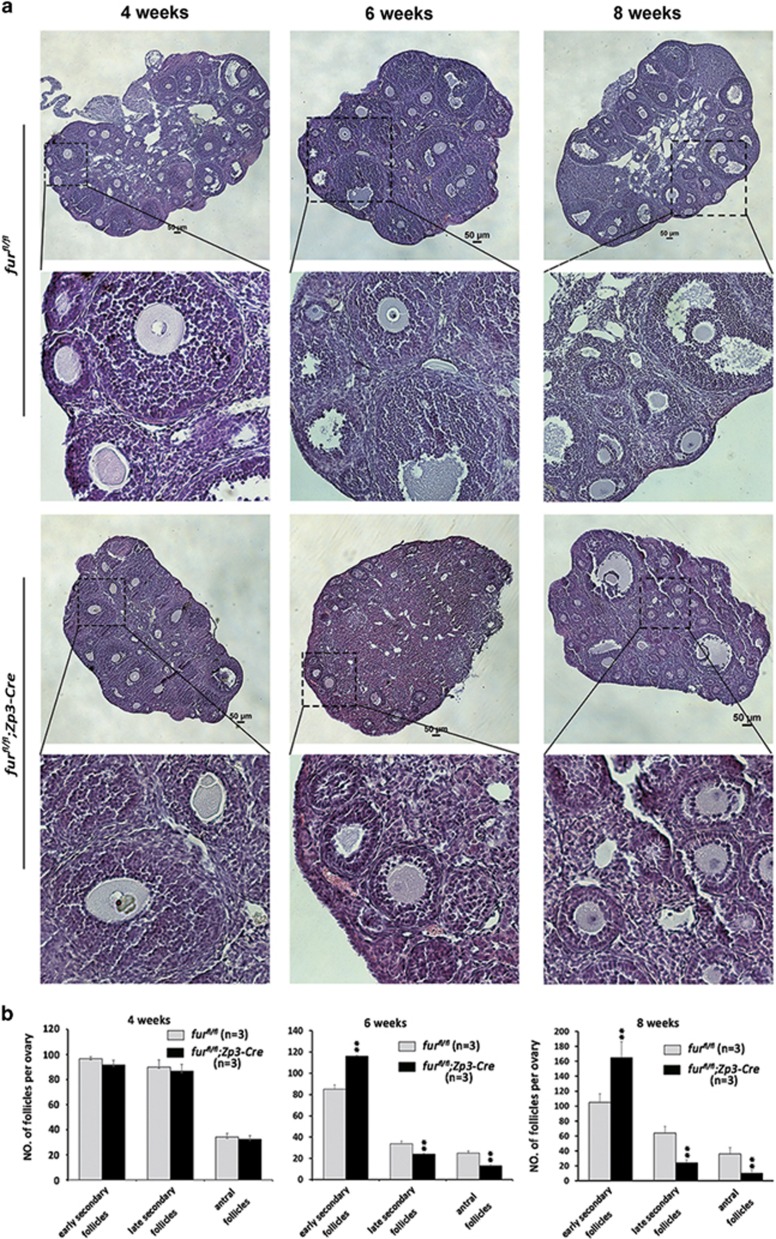
Oocyte-specific deletion of *furin* causes decrease of late growing follicle pool and significant increase of early secondary follicles. (**a**) Histology of ovarian sections from *fur*^*fl/fl*^ and *fur*^*fl/fl*^*;Zp3-Cre* females at 4, 6 and 8 weeks of age, respectively, stained with H&E. Scale bars=50 *μ*m. (**b**) Quantitative analysis of early secondary follicles, late secondary follicles and antral follicles at the age of 4, 6 and 8 weeks, respectively (*n*=3 for each genotype, data are mean±SEM). **P<0.01

The mutant mice showed anovulation in natural ovulation and significantly decreased the number of ovulated oocytes in a superovulation assay after sexual maturation. Histological analysis of ovaries showed that there was no apparent difference in morphology of follicle development in ovaries of *fur*^*fl/fl*^ and *fur*^*fl/fl*^*;Zp3-Cre* mice at 4 weeks (Figure 3a, 4 weeks). However, there was an excessive increase of early secondary follicles and marked loss of large growing follicles in mutant ovaries at 6 weeks of age (Figure 3a, 6 weeks). At the age of 8 weeks, the *fur*^*fl/fl*^*;Zp3-Cre* mice showed significant increase of early secondary follicles as well, and large antral follicles showed degeneration (Figure 3a, 8 weeks). Consistent with these observations, the quantitative analysis showed that the number of early secondary follicles increased significantly, while large growing (late preantral) follicles and antral follicles were remarkably decreased in *fur*^*fl/fl*^*;Zp3-Cre* ovaries compared with the control *fur*^*fl/fl*^ ovaries at the age of 6 weeks (Figure 3b). All the above data demonstrated that the majority of primary follicles could not develop beyond the early secondary follicles in *fur*^*fl/fl*^*; Zp3-Cre* mice by 6 weeks of age.

### Apoptosis and impaired gap junctions between oocytes and surrounded granulosa cells in late growing follicles of mutant mice

**Figure 4:**
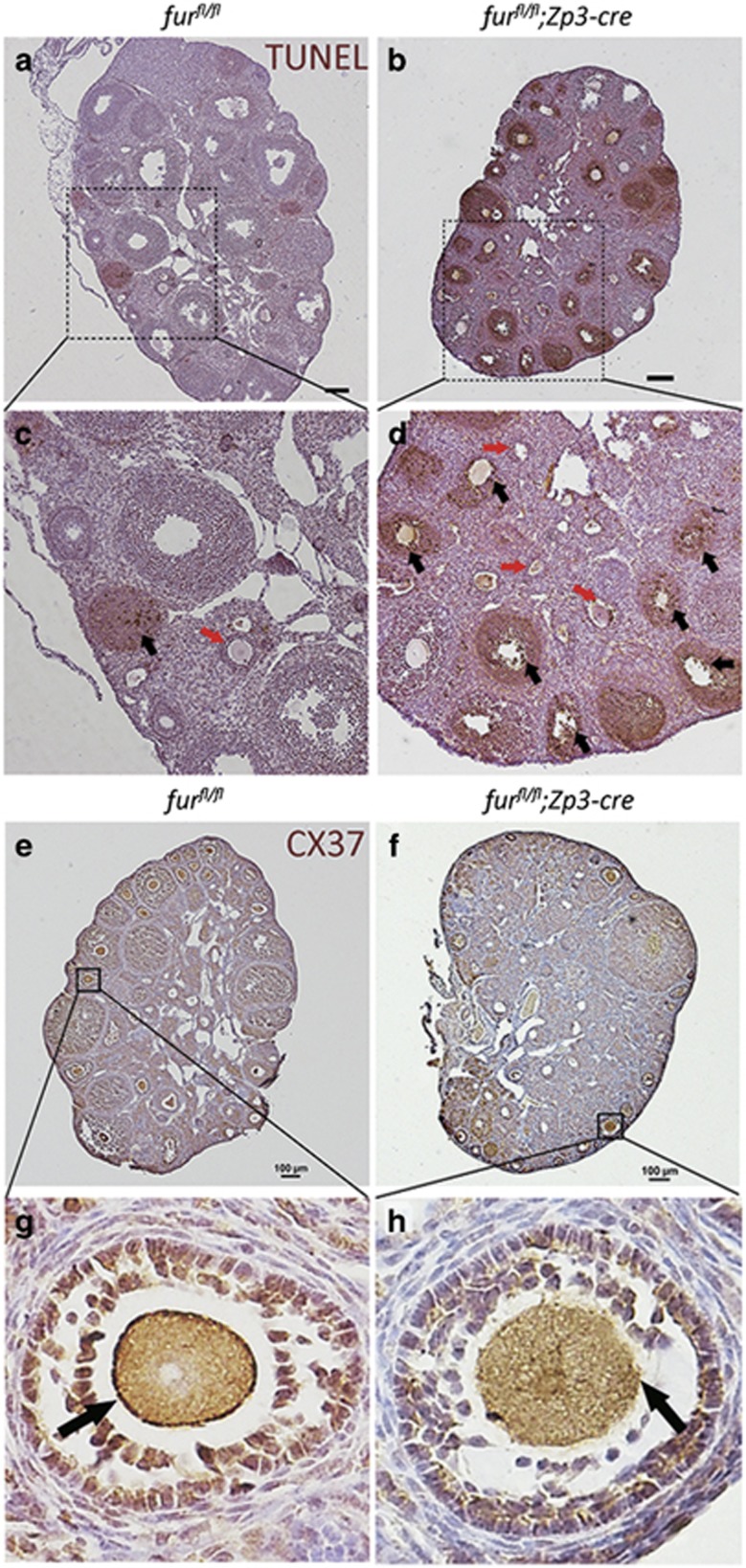
TUNEL assay shows apoptosis in late growing follicles and IHC analysis of connexin 37 shows impaired gap junctions between oocytes and surrounding granulosa cells in mutant mice. (**a**–**d**) TUNEL staining showing apoptotic granulosa cells in the ovaries of *fur*^*fl/fl*^ and *fur*^*fl/fl*^*;Zp3-Cre* at PD 35. Black arrowheads show the apoptosis staining in follicles, and red arrowheads point to the early secondary follicles. Scale bar=50 *μ*m. (**e**–**h**) Connexin 37 (CX37) immunohistochemistry for the ovaries of *fur*^*fl/fl*^ and *fur*^*fl/fl*^*;Zp3-Cre* mice. Black arrowheads show the connexin 37 staining at the surface of oocytes in early secondary follicles. Scale bar=100 *μ*m

At postnatal day 35, we detected severe apoptosis of granulosa cells in late preantral follicles and antral follicles by terminal deoxynucleotidyltransferase-mediated deoxyuridine 5′-triphosphate fluorescein nick-end labeling (TUNEL) assay in *fur*^*fl/fl*^*;Zp3-Cre* ovaries compared with *fur*^*fl/fl*^ ovaries (Figures 4a–d, black arrowheads). However, there was no apparent apoptosis staining in early secondary follicles in *fur*^*fl/fl*^*;Zp3-Cre* ovaries (Figures 4a–d, red arrowheads). Fewer or almost no late growing follicles or antral follicles were observed in *fur*^*fl/fl*^*;Zp3-Cre* ovaries at 8 weeks of age. These data suggested that the *furin* deletion in oocytes led to the atresia of follicles beyond the early secondary stage.

The communication between oocytes and surrounding granulosa cells is achieved by gap junctions, which consists of connexin 37.^30, 31^ We suspected that the gap junctions between oocytes and granulosa cells were impaired in mutant mice. IHC analysis of connexin 37 confirmed this speculation. The connexin 37 showed normal presence in the early secondary follicles in *fur*^*fl/fl*^ ovaries (Figures 4e and g). On the contrary, connexin 37 was almost absent in the early secondary follicles (Figures 4f and h). These results suggested that the gap junctions between oocytes and surrounding granulosa cells were impaired since the early secondary follicle stage.

### Oocyte-specific deletion of *furin* causes the loss of the mature form of ADAMTS1 in oocytes

**Figure 5:**
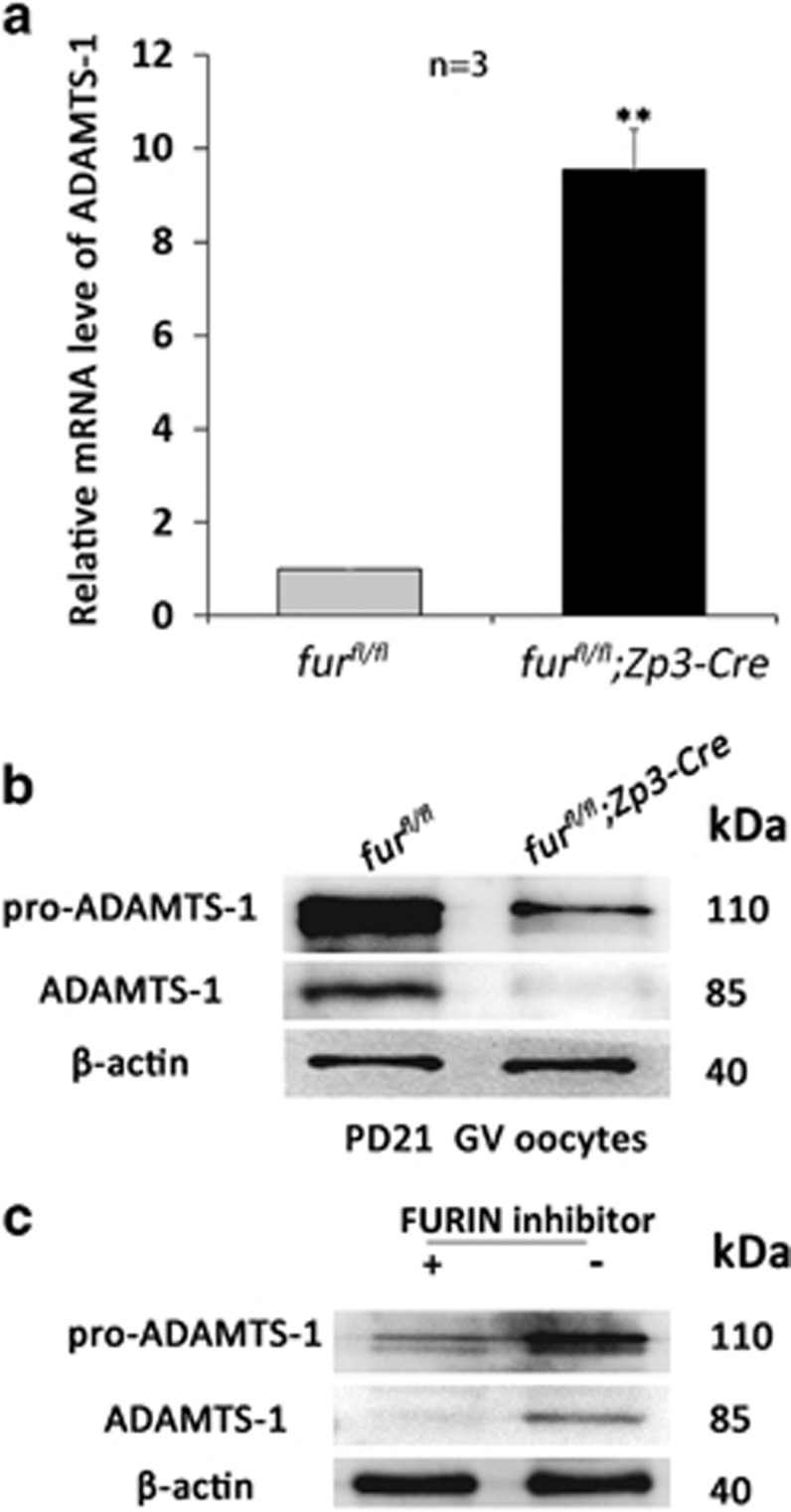
Oocyte-specific deletion of *furin* causes loss of the mature form of ADAMTS1 in oocytes. (**a**) qRT-PCR analysis of the mRNA expression of *ADAMTS-1* in oocytes with the indicated genotypes. **P<0.01 (*n*=3 for each genotype). (**b**) Western blot analysis of corresponding protein level in oocytes from *fur*^*fl/fl*^ and *fur*^*fl/fl*^*; Zp3-Cre* mice. Level of *β*-actin was used as internal controls. Molecular mass was given in kilodaltons. A total of 250 oocytes were used for each lane. For each experiment, at least six mice of each genotype were used. (**c**) Western blot showing defective processing of pro-ADAMTS1 in FURIN inhibitor-treated GV oocytes. Level of *β*-actin was used as an internal control. Molecular mass was given in kilodaltons. A total of 250 oocytes were used for each lane

To investigate the mechanism underlying *furin*-deletion-caused early secondary follicle arrest, we started to investigate the substrates of FURIN based on its function implicated in processing latent precursor proteins into their biologically active products. It has been reported that *ADAMTS-1*-null mutant mice have defects in female fertility and decreased the number of growing follicles.^26, 27^ These results demonstrated that ADAMTS1 is important for follicle development beyond early secondary follicles. Therefore, we determined the mRNA and protein level of *ADAMTS-1* by qRT-PCR and western blot, respectively. *ADAMTS-1* mRNA level increased more than ninefold (Figure 5a); however, the mature form of ADAMTS1 was nearly lost in *fur*^*fl/fl*^*;Zp3-Cre* oocytes (Figure 5b and Supplementary Figure S1A). To investigate whether FURIN is the proprotein convertase responsible for cleavage of pro-ADAMTS1, oocytes were treated with 200 *μ*M decanoyl-l-arginyl-l-valyl-l-lysyl-l-arginyl-chloromethylketone (decanoyl-RVKR-CMK), a peptidyl chloromethylketone that binds to the catalytic site of FURIN to block its activity for 24 h. Consistent with our results *in vivo*, pro-ADAMTS1 cleavage was efficient in untreated oocytes. However, oocytes treated with decanoyl-RVKR-CMK showed complete inhibition of FURIN activity and only pro-ADAMTS1 was detected (Figure 5c and Supplementary Figure S1B). These data suggest that the phenotype of *fur*^*fl/fl*^*;Zp3-Cre* might have resulted from the lack of mature ADAMTS1.

### Deletion of *furin* in oocytes does not affect the activation of primordial follicles

**Figure 6:**
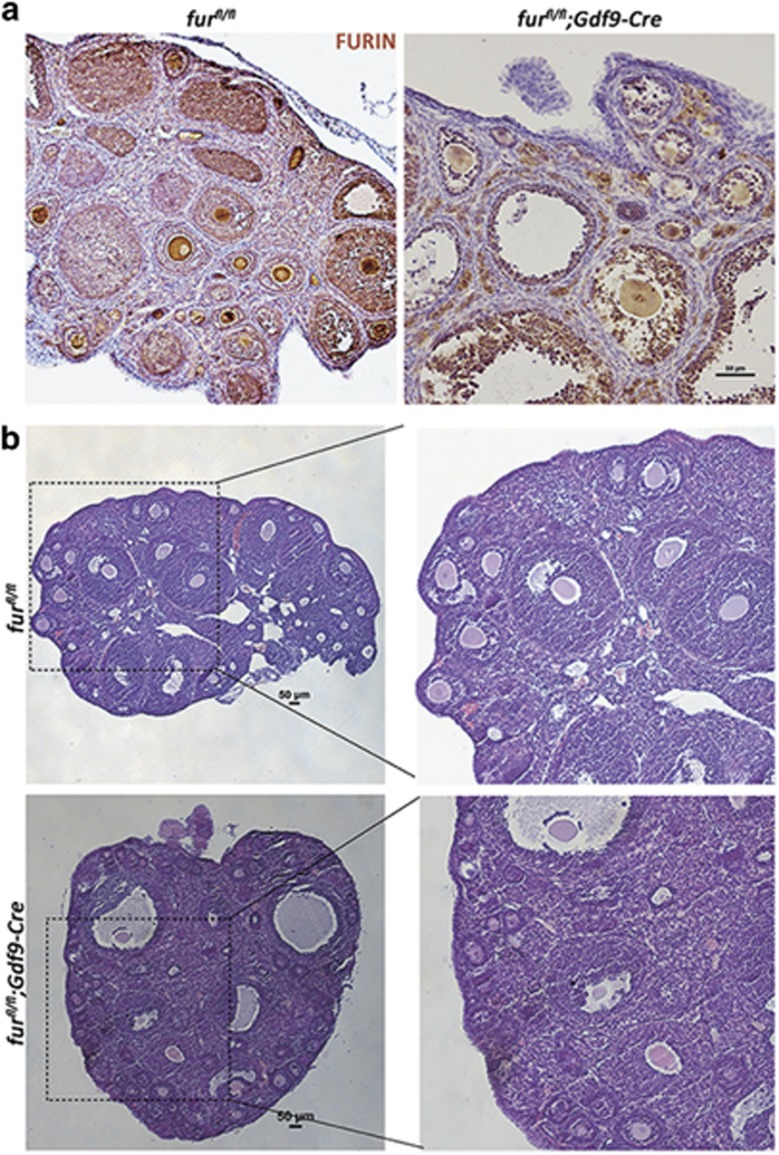
Deletion of *furin* in primordial follicle oocytes causes early secondary follicle developmental arrest. (**a**) FURIN immunohistochemistry showing specific FURIN protein depletion in the oocytes of *fur*^*fl/fl*^ and *fur*^*fl/fl*^*; Gdf9-Cre* mice. Scale bar=50 *μ*m (**b**) Histology of ovarian sections from *fur*^*fl/fl*^ and *fur*^*fl/fl*^*;Gdf9-Cre* 6-week-old females stained with H&E. Scale bars=50 *μ*m

**Figure 7:**
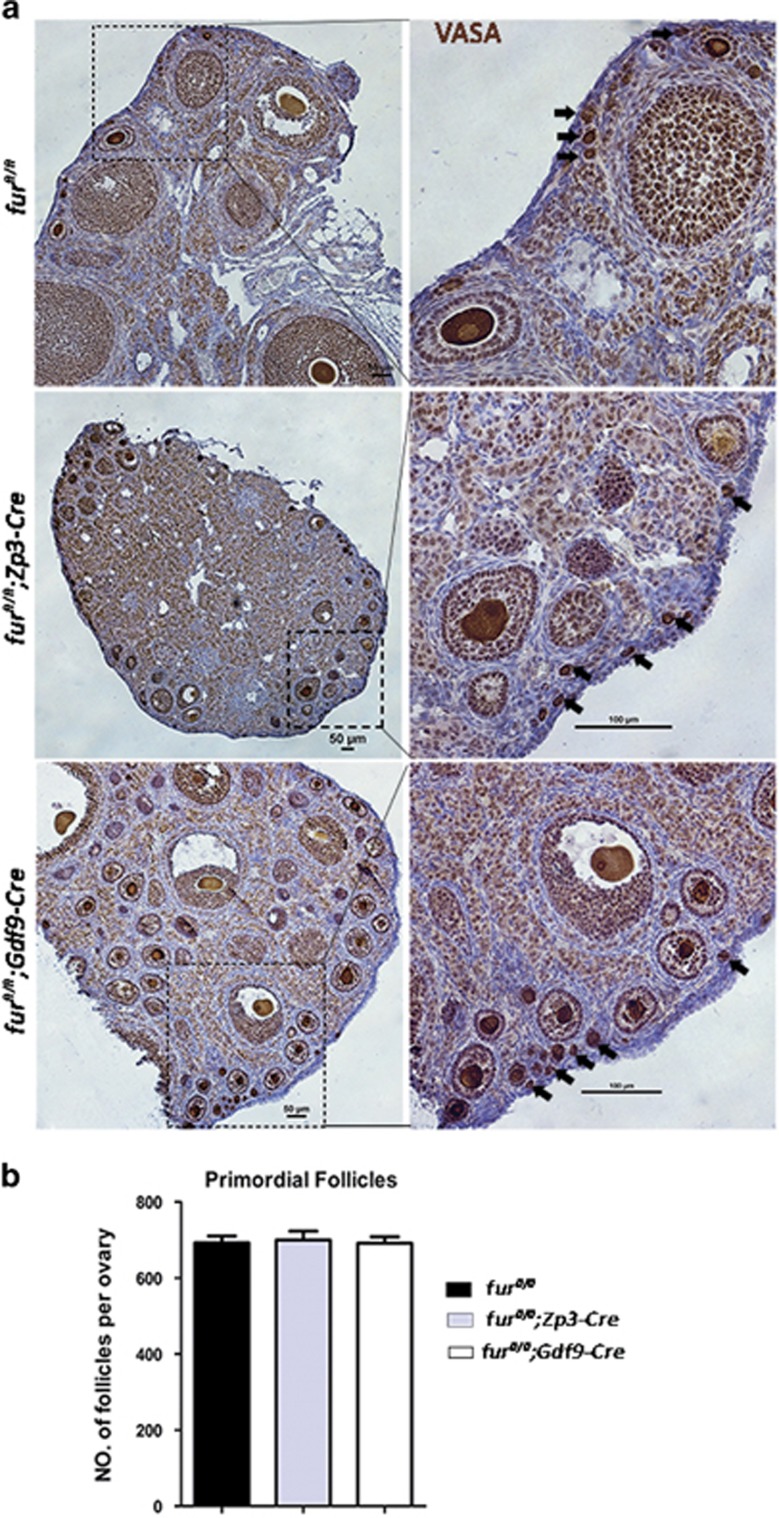
Deletion of *furin* in oocytes does not affect the activation of primordial follicles. (**a**) Mouse VASA homolog (MVH) IHC staining showing normal primordial follicle pool in 2-month-old *fur*^*fl/fl*^*;Zp3-Cre* and *fur*^*fl/fl*^*;Gdf9-Cre* female mice as well as *fur*^*fl/fl*^ female mice. Black arrowhead shows the primordial follicles. Scale bar=50 *μ*m. (**b**) Shown are the quantifications of the number of primordial follicles per ovary at the age of 2 months. Data are shown as mean±SEM. For each experiment at least five mice of each genotype were used

In female *fur*^*fl/fl*^*;Zp3-Cre* mice, when the *furin* gene is targeted in oocytes of primary follicles, the follicles are arrested at the early second follicle stage. Thus, we wondered whether FURIN had roles in primordial follicle activation. We generated *fur*^*fl/fl*^*;Gdf9-Cre* female mice by crossing *fur*^*fl/fl*^ with *Gdf9-Cre* transgenic mice to identify whether disruption of FURIN in oocytes had an effect on primordial follicles. IHC analysis of FURIN showed the successful deletion of *furin* in oocytes (Figure 6a). In accordance with infertility in *fur*^*fl/fl*^*;Zp3-Cre* mice, the *fur*^*fl/fl*^*;Gdf9-Cre* female mice were also infertile. Histological analysis of ovaries in *fur*^*fl/fl*^*;Gdf9-Cre* mice showed apparent increase in early secondary follicles at 6 weeks of age, which was the same as the phenotype in *fur*^*fl/fl*^*;Zp3-Cre* mice (Figure 6b). We then performed immunostaining for the germ cell marker mouse VASA homolo) on 2-month-old ovarian sections. As shown in Figure 7a, primordial follicles were mostly scattered around the cortical region in *fur*^*fl/fl*^*;Gdf9-Cre* ovaries as well as in *fur*^*fl/fl*^ and *fur*^*fl/fl*^*;Zp3-Cre* mice. Consistent with immunohistological staining, the quantitative analysis demonstrated that there was no difference in the number of primordial follicles in the ovaries, where all the genotypes contained almost the same amount of primordial follicles (Figure 7b). These results suggested that deletion of *furin* in oocytes did not have an evident influence on the survival and activation of primordial follicles in ovaries of *fur*^*fl/fl*^*;Zp3-Cre* mice and in *fur*^*fl/fl*^*;Gdf9-Cre* mice. The early secondary follicle arrest was not caused by excessive activation of primordial follicles.

### The proliferation of granulosa cells is compromised in mutant mice

**Figure 8:**
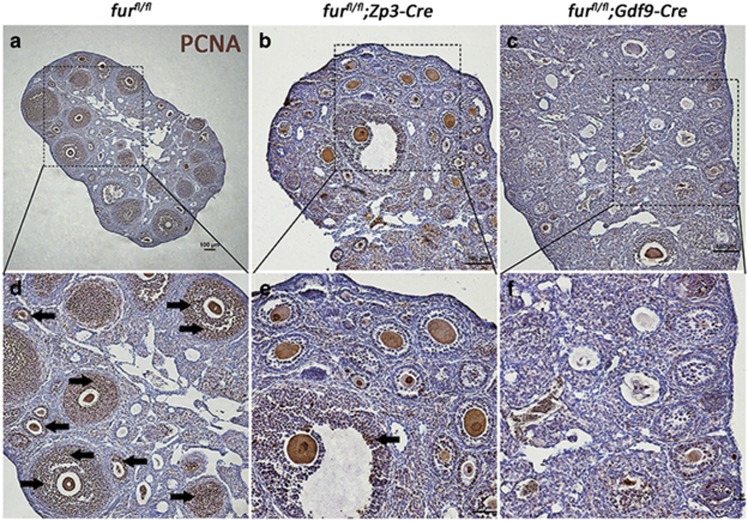
The proliferation of granulosa cells is compromised in mutant mice. Proliferating cell nuclear antigen (PCNA) immunohistochemistry showing the status of granulosa cell proliferation in follicles. Black arrowheads point to the PCNA staining in granulosa cells in early secondary follicles. Scale bar=100 *μ*m

Given the impaired gap junctions between oocytes and surrounding granulosa cells in mutant mice, we suspected that *furin* deletion from oocytes might impact the proliferation of granulosa cells. To test this, we performed immunostaining for cell proliferation marker PCNA in 2-month-old ovarian sections. As shown in Figure 8a, in *fur*^*fl/fl*^ ovaries, PCNA staining was present throughout the granulosa cell layers in the control group; in contrast, PCNA staining was nearly absent in the ovaries of *fur*^*fl/fl*^*; Zp3-Cre* mice (Figure 8b) and *fur*^*fl/fl*^*; Gdf9-Cre* mice (Figure 8c). These data demonstrated that there was a profound defect in granulosa cell proliferation in *fur*^*fl/fl*^*; Zp3-Cre* mice and in *fur*^*fl/fl*^*;Gdf9-Cre* mice, which might lead to the early secondary follicles arrest.

## Discussion

Follicular recruitment, frequently used by different investigators to describe two important points during follicular development, includes initial recruitment and cyclic recruitment.^32^ The primordial follicles are continuously recruited into the growing follicle pool during initial recruitment, whereas a cohort of follicles is recruited to develop into antral follicles per menstrual cycle. The initial recruitment of primordial follicles and the development of secondary follicles to the antral stage are critical during folliculogenesis.^7^ Although the former was recently intensively investigated by others and us,^4, 6, 33, 34, 35^ the mechanism involved in the subsequent development of early secondary follicles remained poorly understood. Ogiwara *et al.*^36^ reported the greatest expression of *furin* mRNA in oocytes of small growing follicles compared with large growing follicles and antral follicles, indicating that FURIN might have an important role in further development of small growing follicles. In the present study, by using a mutant mouse model with oocyte-specific deletion of *furin*, we demonstrated that FURIN is essential for follicular development beyond the early secondary follicles.

To explore the reason for the rapid decrease of late growing follicles from PD30 to PD42, we conducted an apoptosis test. The TUNEL staining showed that the large follicles beyond the early secondary follicles were induced to undergo apoptosis, suggesting a defective effect in those follicles. Furthermore, the gap junctions between oocytes and surrounding granulosa cells were impaired from the early secondary follicles, which may affect the normal follicular development beyond the early secondary follicles.

Although the apoptosis staining explained the decreased number of large follicles, it could not account for the increased number of early secondary follicles. To investigate the mechanisms underlying the developmental arrest of early secondary follicles, we designed experiments to find potential molecules responsible for this phenotype. It has been reported that FURIN is involved in processing latent precursor proteins into their biologically active products. It has further been reported that female *ADAMTS-1*-null mutant mice have severe defects in fertility and significantly decreased the number of late growing follicles, suggesting that ADAMTS1 is important for follicle development beyond the type 4 and/or type 5a (early secondary follicles).^27^ Interestingly, ADAMTS1 is synthesized as pro-ADAMTS1. In accordance with *ADAMTS-1*-null phenotype in the ovary, oocyte-specific ablation of FURIN caused the developmental arrest of early secondary follicles. Based on this, we determined whether the mRNA and protein level of *ADAMTS-1* was affected in oocytes in mutant mice. Surprisingly, although the *ADAMTS-1* mRNA level was increased up to ninefold, the mature ADAMTS1 protein was nearly lost upon the disruption of FURIN in oocytes of mutant mice. Therefore, we suggest that a defect in the mature ADAMTS1 could be the most probable cause. In addition, we should note that there is no accumulation of pro-ADAMTS1. In fact, we did not know the reason why there is no accumulation of pro-ADAMTS1 and even decrease. To our knowledge, Louagie *et al.*^19^ investigated the role of FURIN in the endocrine pancreas by conditional knockout model and found that there was no accumulation of proinsulin II and pro-PC2 in spite of impaired processing of proproteins. We suggest that there might be one possible mechanism for control of the translation efficiency *in vivo*, which remains to be studied.

To investigate whether ablation of FURIN also affects the primordial follicles recruited into the growing follicle pool during the initial recruitment, we generated *fur*^*fl/fl*^*;Gdf9-Cre* female mice to achieve deletion of *furin* from primordial follicles. The *fur*^*fl/fl*^*;Gdf9-Cre* female mice were also infertile and showed developmental arrest of early secondary follicles, similar to the phenotype in *fur*^*fl/fl*^*;Zp3-Cre* female mice. The IHC staining of the germ cell marker VASA confirmed that there was no influence on the primordial follicle pool.

Normal communication between oocytes and surrounding granulosa cells as well as the granulosa cell proliferation are indispensable for normal folliculogenesis. ADAMTS1 is implicated in many physiological events, such as angiogenesis inhibition, various inflammatory processes and development of cancer cachexia. It has been reported that *ADAMTS-1*-null mice showed female infertility and impaired follicular development.^26, 27^ Subsequently, Brown *et al.*^37^ demonstrated that ADAMTS1 was required for extracellular matrix (ECM) remodeling during ovarian follicular development and formation of lymphatic vessels.^38^ In accordance with this, Russell *et al.*^39^ showed that pro-ADMATS1 was localized to cytoplasmic secretory vesicles and the mature form of ADAMTS1 was accumulated in ECM. Particularly, it has be demonstrated that ADAMTS1 is an ECM-bound metalloproteinase,^26^ and ECM components and ECM-associated molecules, such as proteoglycans, aggrecan and versican, are targets of ADAMTS1.^40–42^ Meanwhile, Horiguchi *et al.*^43^ reported that ECM component laminin promoted the formation of gap junction in the rat anterior pituitary gland, which inspired us to investigate the gap junction between oocytes and granulosa cells. IHC analysis of connexin 37 showed impaired gap junctions between oocytes and granulosa cells. IHC staining of the cell proliferation marker PCNA indicated that the proliferation of granulosa cells was compromised, which might partially explain the developmental arrest of early secondary follicles.

In summary, FURIN is indispensable for female fertility and FURIN-ADAMTS1 is potentially partnering as an enzyme substrate in oocytes, which may be responsible for follicular development beyond the early secondary follicles. Our study may provide an important *in vivo* model for further understanding the mechanism of early-stage follicular development.

## Materials and methods

### Mice

The previously described *fur*^*fl/f*^*^l^* mice, maintained with a mixed genomic background of 129S4/SvJae and C57BL/6 J (The Jackson Laboratory, Laboratory of John W.M. Creemers, KU Leuven, Belgium; 016913), were crossed with B6J/CBAJ *Zp3-Cre* transgenic mice and B6J/CBAJ *Gdf9-Cre* transgenic mice. Mutant mice were homozygous for the FURIN-floxed allele and heterozygous for *Zp3-Cre* (*fur*^*fl/fl*^*;Zp3-Cre*) or for *Gdf9-Cre*(*fur*^*fl/fl*^*;Gdf9-Cre*), respectively. Control mice were homozygous for FURIN-floxed allele (*fur*^*fl/fl*^) without Zp3-cre or Gdf9-Cre. Mice were housed in 12-h alternating light–dark cycles, with free access to water and food.

All experiments were conducted under the approval of the Animal Research Committee of the Institute of Zoology, Chinese Academy of Sciences, China. Mice were killed under standard protocols, and all efforts were made to minimize suffering.

### Fertility assessment, natural ovulation and superovulation analysis

To assess the reproductive activity, seven individually housed control or mutant mice were mated with a male mouse validated to be fertile at the age of 6 weeks. Cages were examined two times a week and the number of pups was recorded up to 6 months. For the natural ovulation assay, 6–9-week-old female mice were examined for the estrous cycle every day. Females in the estrus were mated with a fertile male. The next day in the morning, the oviducts were dissected from the female with the plug, and zygotes were collected and counted. To induce synchronized follicular growth and ovulation, each 6–9-week-old female mouse was injected with 10 IU PMSG, followed by 10 IU hCG after 48 h to promote ovulation. The oviducts were dissected and cumulus–oocyte complexes were collected at 16 h of hCG treatment. After a 3- min treatment with 0.5 mg/ml hyaluronidase (Sigma, St. Louis, MO, USA) in the M2 medium, oocytes were collected, counted and then analyzed.

### Oocyte collection and culture

The GV oocytes were isolated from ovaries of 3- to 4-week-old female mice. MII stage oocytes were isolated from 3- to 6-week-old female mice. Oocytes were cultured in the M2 medium (Sigma) under paraffin oil at 37 °C with 5% CO_2_ in air.

### Histological analysis and IHC staining of ovary

Ovaries were fixed in 4% paraformaldehyde (pH 7.5) overnight at 4 °C, dehydrated in 50%, 70%, in a graded ethanol series, cleared in xylene three times and finally embedded in paraffin wax. The treated ovaries were sectioned consecutively at 8 *μ*m for hematoxylin and eosin (H&E) staining and IHC staining. Serial ovarian sections were photographed and follicles were counted in every fifth sections using the Image J software and cell counter plugin.

### Quantification of ovarian follicles

Quantification of ovarian follicles was performed as previously described by Liu *et al.*^4^ Briefly, to count the number of follicles, paraffin-embedded ovaries were serially sectioned at 8-*μ*m thickness and every fifth section was mounted on slides. Then, these sections were stained with H&E for morphological analysis. Ovarian follicles at different developmental stages, including primordial follicles, primary follicles (type 3), early secondary and late secondary follicles (type 4 and type 5) and antral follicles (type 6 and type 7) were counted in collected sections of an ovary, based on the well-accepted standards established by Pederson and Peters.^5^ In each section, only those follicles in which the nucleus of the oocyte was clearly visible were scored and the cumulative follicle counts were multiplied by a correction factor of 5 to represent the estimated number of total follicles in an ovary.

### Quantitative real time-PCR

RNA was isolated from ~100 oocytes for each group and reverse transcription reaction was carried out using the RNeasy Kit (Qiagen, Hilden, Germany). mRNA level of each gene was validated by qRT-PCR analysis (Roche; 480, Boehringer, Mannheim, Germany) according to the manufacturer's instruction. Primers were the following: FURIN-qF (forward primer): 5′-CAGAAGCATGGCTTCCACAAC-3′ FURIN-qR (reverse primer): 5′-TGTCACTGCTCTGTGCCAGAA-3′ ADAMTS1-qF (forward primer): 5′-CATAACAATGCTGCTATGTGCG-3′ ADAMTS1-qR (reverse primer): 5′-TGTCCGGCTGCAACTTCAG-3′ *β*-actin-qF: (forward primer): 5′-GGCTGTATTCCCCTCCATCG-3′ *β*-actin-qR: (reverse primer): 5′-CCAGTTGGTAACAATGCCATGT-3′.The experiments were repeated at least three times.

### TUNEL assay

Analysis of apoptosis in ovarian follicles was carried out by TUNEL assay using the ApopTag Plus *In Situ* Apoptosis Detection Kit (Chemicon International, Temecula, CA, USA). At least four different specimens from each genotype were analyzed in parallel.

### FURIN-specific inhibition

Decanoyl-RVKR-CMK was obtained from Merck Millipore (no. 344930, USA). The FURIN inhibitor was dissolved in dimethyl sulfoxide at a concentration of 25 mM, and this stock solution was added directly to the M2 medium. The GV oocytes were treated with 200 *μ*M decanoyl-RVKR-CMK for 24 h.

### Western blot analysis

A total of 200 oocytes per sample were mixed with 2 × SDS sample buffer and boiled for 5 min at 100°C for SDS-PAGE. Western blotting was performed as described previously,^44^ using the primary antibody dilution anti-FURIN (Abcam, Cambridge, MA, USA) at 1:1000; anti-ADAMTS1 (MAB1810, Merck Millipore, USA) at 1:1000; anti-*β*-actin (Zhongshan Golden Bridge Biotechnology, Beijing, China) at 1:1000. Horseradish peroxidase-linked secondary antibodies (Zhongshan Golden Bridge Biotechnology) were diluted at 1:2000. Protein bands were detected using Thermo Supersignal West Pico chemiluminescent substrate.

### Statistical analysis

All data presented were collected in at least three independent experiments and analyzed using SPSS (SPSS, IBM, USA). Data were expressed as mean±S.E.M. and significance of differences was evaluated with Student’s *t*-test.
