# Supplementary figures and images for: Oocyte-specific deletion of furin leads to female infertility by causing early secondary follicle arrest in mice
Source: Cell Death Dis. 2017 Jun 1;8(6):e2846–. doi: 10.1038/cddis.2017.231 (PMC5520891; doi:10.1038/cddis.2017.231)

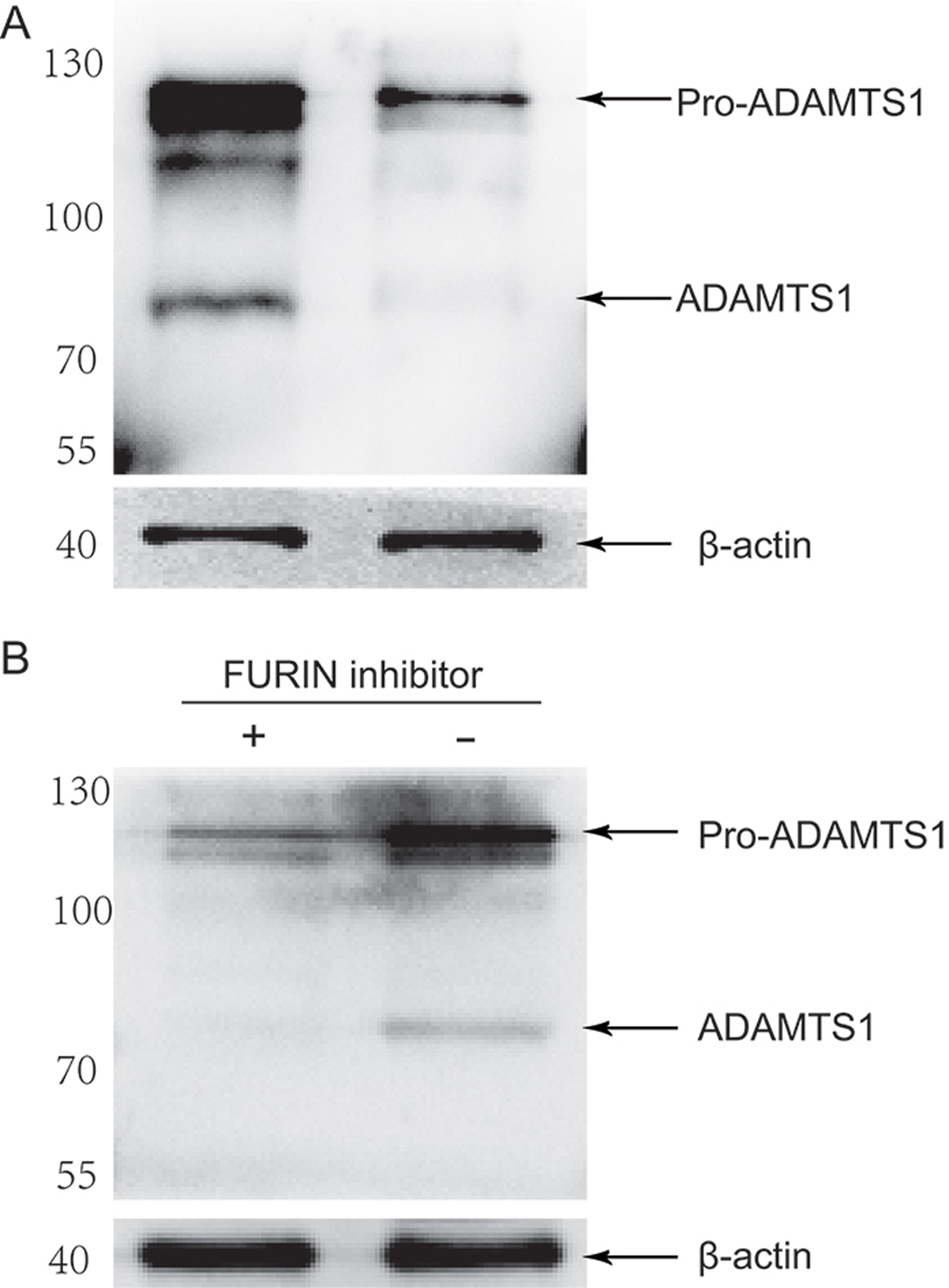

Supplement: Supplementary Figure [file cddis2017231x1.tif]
